# The influence of environmental variables on platelet concentration in horse platelet-rich plasma

**DOI:** 10.1186/s13028-016-0226-3

**Published:** 2016-07-04

**Authors:** Riccardo Rinnovati, Noemi Romagnoli, Fabio Gentilini, Carlotta Lambertini, Alessandro Spadari

**Affiliations:** Department of Veterinary Medical Sciences, University of Bologna, Via Tolara di Sopra 50, 40064 Ozzano Dell’Emilia, BO Italy

**Keywords:** Double transfusion bags, Environmental variables, Horse, Platelet concentration, Platelet-rich plasma

## Abstract

Platelet-rich plasma (PRP) commonly refers to blood products which contain a higher platelet (PLT) concentration as compared to normal plasma. Autologous PRP has been shown to be safe and effective in promoting the natural processes of soft tissue healing or reconstruction in humans and horses. Variability in PLT concentration has been observed in practice between PRP preparations from different patients or from the same individual under different conditions. A change in PLT concentration could modify PRP efficacy in routine applications. The aim of this study was to test the influence of environmental, individual and agonistic variables on the PLT concentration of PRP in horses. Six healthy Standardbred mares were exposed to six different variables with a one-week washout period between variables, and PRP was subsequently obtained from each horse. The variables were time of withdrawal during the day (morning/evening), hydration status (overhydration/dehydration) treatment with anti-inflammatory drugs and training periods on a treadmill. The platelet concentration was significantly higher in horses treated with a non-steroidal anti-inflammatory drug (*P* = 0.03). The leukocyte concentration increased 2–9 fold with respect to whole blood in the PRP which was obtained after exposure to all the variable considered. Environmental variation in platelet concentration should be taken into consideration during PRP preparation.

## Findings

Platelet-rich plasma (PRP) is a suspension of platelets, white blood cells (WBCs) and a minimal number of red blood cells in a small volume of plasma. The ideal concentration of cellular components in PRP products has not yet been established and is the topic of ongoing research and debate [1–3].

The currently accepted benchmark for PRP used in human medicine is a platelet (PLT) concentration of at least a four/five-fold increase over whole blood or a platelet count range between 150 × 10^9^/l and 350 × 10^9^/l [2, 4]. In horses, where the baseline concentration of platelets is 200 × 10^9^/l [5], a benchmark of 250 × 10^9^/l has commonly been used for equine PRP [6].

The preparation method may significantly affect the concentrating capabilities of some PRPs [6]. The benefits of a high PLT concentration in PRP (4358 ± 265 × 10^9^/l) to stimulate bone regeneration in human medicine has been documented [7].

The variability in the PLT concentration between preparations from different patients or from the same individual under different conditions could hamper the establishment of the most effective PLT concentration and PLT/WBC ratio in experimental studies, or reduce the PRP efficacy in routine applications. In an animal model using rabbits, the hematocrit, among other haematological variables, was found to be the main hematological parameter influencing the PRP concentration [8]. In horses, “intrinsic” individual factors, such as breed, age and gender, have been demonstrated to play a role in PRP composition [1]. No data have been reported regarding the hematological parameters influencing PRP composition in horses.

The present study was designed to analyse some environmental and intrinsic factors which could influence the PRP platelet concentration in equine medicine. The following parameters were considered: time of day, overhydration, dehydration, anti-inflammatory drug (NSAID) therapy and exercise. This study was approved by the ethics committee of the University of Bologna in accordance with European Economic Community Council Directive 86/609, adopted by the Italian Government (D.L. 27/01/1992 no. 116).

Six healthy Standardbred mares were used for the study. The median age of the horses was 16 years (12–18 years) and the median weight was 480 kg (range 370–520 kg).

The entire protocol was carried out over nine weeks. Every week, each horse was scheduled randomly for one variable with respect to the others. The variables were:

*Time of day* the blood sampling was carried out at 7.00 a.m. (DAY) and at 7.00 p.m. (NGH). The platelet and the WBC data obtained from the hematological evaluation before any treatment were considered to be the control PLT and the WBC blood values (Table 1).

**Table 1:** Platelet concentration and white blood cell count

Platelet and WBC concentration count ( 10^9^/L )				
Group	Platelet in whole blood median	Platelet in whole blood range	WBC in whole blood median	WBC in whole blood range
DAY	149	119–156	8.605	7.21–10.53
NGH	133	106–187	8.81	6.71–9.38
HYD	100	70–190	7.195	5.81–7.67
DHY	138	51–227	8.58	7.34–9.97
NSAID	108	65–171	8.71	6.18–9.86
EXE	129	78–143	9.6	6.69–10.51

Summary of the platelet concentration and white blood cell (WBC) count in whole blood in the different groups

Group: Time of day: The blood sampling was carried out at 7.00 a.m. (DAY) and at 7.00 p.m. (NGH); overhydration (HYD): the blood sampling was carried out before and after an overload of Ringer Lactate; dehydration (DHY): the blood sampling was carried out before and after withholding water; non-steroidal anti-inflammatory (NSAID) drug therapy: the blood sampling was carried out before and after anti-inflammatory therapy; exercise (EXE): the blood sampling was carried out before and after standardised exercise training

*Overhydration (HYD)* Blood sampling was carried out before and after an overload of Ringer Lactate; intravenous administration was stopped when the urine specific gravity was equal to or less than 1020.

*Dehydration (DHY)* Blood sampling was carried out before and after withholding water until an increase in serum creatinine and serum albumin above our laboratory reference interval (RI) [creatinine RI: 0.90–1.50 mg/dL; albumin RI: 2.90–3.70 g/dL] was observed.

*NSAID drug therapy* Blood sampling was carried out before and after anti-inflammatory therapy with ketoprofen (2 mg/kg intravenously) every 24 h for 5 days.

*Exercise (EXE)* Blood sampling was carried out before and after standardised exercise training consisting of 3 min at 2 m/s followed by 3 min at 4 m/s, and then 3 min at 6 m/s on a treadmill. This program was carried out for 9 days; on the 10th day, a speed test was carried out by adding 3 min at a speed of 8 m/s to the previous protocol.

Blood was collected in the morning (unless indicated differently) with a 1 week washout interval between experiments (washout period exceeding the detection time for Fédération Equestre Internationale of 96 h). Each of the six horses underwent each treatment randomly. Before the first PRP processing, a 1 ml aliquot of whole blood was saved for analysis of baseline values.

In each horse, 450 ml of blood was harvested from the jugular vein and was stored in double transfusion bags containing a citrate–phosphate-dextrose solution with adenine (CPDA-1) (Terumo BCT, Roma, Italy). The separation of the blood cells was carried out within 3 h after the sampling using a refrigerated laboratory centrifuge (Heraeus Cryofuge 6000, Thermo scientific, Waltham, USA). Centrifugation was first performed at 350*g* for 20 min at 22 °C in order to obtain separation of the erythrocytes and the WBCs from the plasma and the platelets. The satellite bag underwent a second centrifugation at 3300*g* for 10 min at 22 °C to obtain PRP, as a turbid fraction, and platelet-poor plasma. The PRP was transferred to a 1.5 ml tube under sterile conditions.

Data are reported as medians and ranges. In the variable groups, the PLT and the WBC concentrations were compared using the base values (whole blood) and the concentrations obtained after exposition to the variables; moreover, a comparison between the groups was carried out using a Wilcoxon paired sample test (MedCalc 6.3). The results were considered statistically significant for *P* < 0.05.

**Fig. 1:**
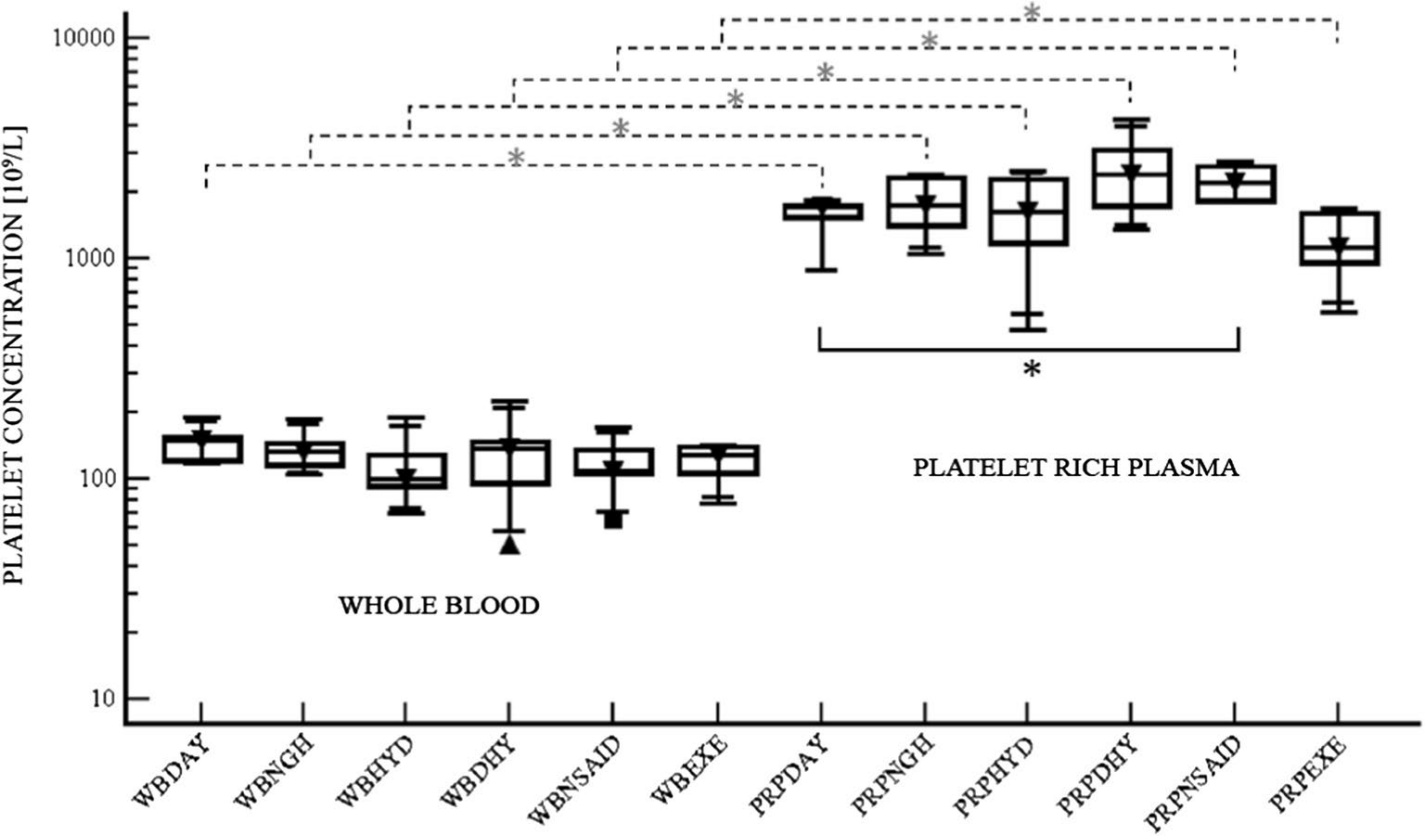
Platelet concentration. *Box plots* of platelet concentration [10^9^/l] in whole blood (WB) and platelet rich plasma (PRP) in each treatment group. The samples were obtained in each horse (six animals) at 7.00 a.m. (DAY) and at 7.00 p.m. (NGH), before and after an overload of Ringer Lactate (HYD), before and after withholding water (DHY), before and after treatment with non-steroidal anti-inflammatory drugs (NSAIDs), and before and after exercise training (EXE). *Box plots* show the median, upper and lower interquartile and range. The *dashed lines* represent the comparison between WB and PRP. The continuous line represents the pairwise correlations between the DAY group (control group) and the NSAID group. *(*P* = *0.03*)

**Fig. 2:**
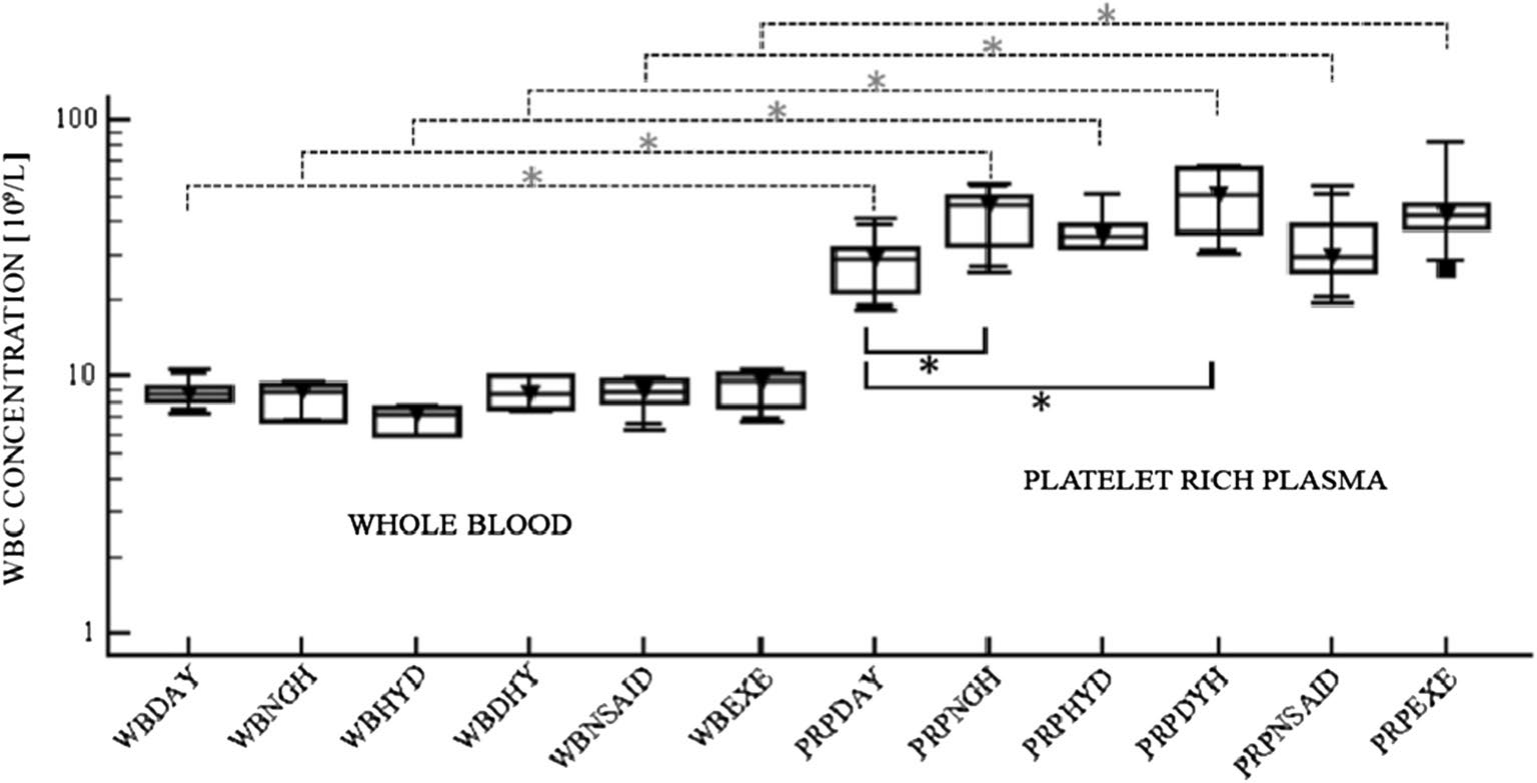
White blood cell concentration. *Box plots* of white blood cell (WBC) concentration [10^9^/L] in whole blood (WB) and platelet rich plasma (PRP) in each treatment group. The samples were obtained in each horse (six animals) at 7.00 a.m. (DAY) and at 7.00 p.m. (NGH), before and after an overload of Ringer Lactate (HYD), before and after withholding water (DHY), before and after treatment with non-steroidal anti-inflammatory drugs (NSAID), and before and after exercise training (EXE). *Box plots* show the median, upper and lower interquartile and range. The *dashed lines* represent the comparison between WB and PRP (*P = 0,03). The continuous line represents the pairwise correlations between the DAY group (control group), and the NGH group *(*P* = *0.03*) and DHY group *(*P* = *0.02*)

As expected, the PRP preparation produced significantly increased levels of both the PLT and the WBC concentrations as compared to whole blood after exposure to all the variables. (Figures 1, 2).

The platelet concentration in the PRP was significantly higher in the horses treated with NSAIDs as compared to the DAY group (*P* = 0.03) (Fig. 1). In all the PRPs obtained after exposure to the variables, the leukocyte concentration increased 2–9 fold with respect to the whole blood value. A higher WBC concentration was measured in the PRP of the DHY group as compared with the DAY group (Fig. 2) and this difference was statistically significant (*P* = 0.02).

The results demonstrated that the PLT concentration in PRP was significantly higher in horses treated with NSAIDs as compared to the DAY group. However, it was not possible to find any significant differences associated with the other variables. A 1 week washout period reduced the risk of the interference of residual drug in the blood before exposure to next variable.

A difference was detected between the DAY and the NGH sampling in the WBC concentration in the PRP (*P* = 0.03). Clinicians should be aware that the effects of PRP are based not solely on PLT concentration, even though many of the reparative factors are contained in the PLTs. The optimal combination of each cellular component in the PRP remains unknown and, since recent studies do not report ideal PLT or WBC concentrations, it is unknown which PRP preparation is best for each clinical indication [9].

This study demonstrated that the use of ketoprofen might affect the PLT concentration in PRP, probably due to the decreased platelet aggregation, as with other NSAIDs [8, 10]. Indeed, PLT aggregates have a higher sedimentation rate with respect to isolated PLTs leading to a position closer to the cell pellet after centrifugation. This may cause a depletion of PLTs from the plasmatic component. It would be of pivotal importance to assess whether the reduced aggregation ability of the PLTs could further affect the release of the mediators and factors necessary for the PRP to be effective. The NSAID variable (ketoprofen), was considered due to its frequent use in horses. During PRP preparation, the presence of NSAIDs in the blood would prevent the production of tromboxane A2 by platelets since it is a strong platelet aggregating factor. This information is crucial in order to know whether or not to suspend the NSAID treatment before PRP production. Different factors have been reported to influence the platelet concentration. In particular, the hematocrit has been found to reduce the platelet concentration [8] while, in female horses, the PLT concentration was higher than in male horses [1]. In the current study, six adult female Standarbred horses were chosen in order to reduce the number of intrinsic factors which could have influenced the results.

Optimisation of the PLT preparation should include understanding the role of WBCs regarding the clinical effects attributed to PRP [11]. Leukocytes contain and produce biologically active catabolic or inflammatory cytokines which could influence the clinical outcome of a PRP application. Moreover the concentration of tumor necrosis factor alpha and interleukin-1-beta seems to be correlated with the neutrophil number in PRP [12].

Recent studies have demonstrated that WBC reduction is important for the success of PRP in some musculoskeletal applications [3, 13]. Our study demonstrated that the WBC concentration of the PRP of the DHY group was higher than the concentration found in the DAY group (*P* = 0.02). According to our experience and based upon the information available, dehydration should be corrected before blood withdrawal for PRP preparation in order to avoid increased WBC content in the PRP.

Our study points out the fact that, in clinical studies involving PRP preparations, it is important to take environmental factors into consideration as they may impact the expected PLT and WBC concentrations. In fact, both components could be influenced by individual external conditions in PRP preparations. Additional studies are necessary to investigate other variables and to confirm the results obtained for ketoprofen, also in sires/geldings, in other age groups and with other NSAIDs in order to definitively strengthen the conclusions of this study.

## Abbreviations

CPDA: a citrate–phosphate-dextrose solution with adenine
DHY: dehydration
EXE: exercise
HYD: overhydration
NSAID: non-steroidal anti-inflammatory drugs
PLT: platelets
PRP: platelet-rich plasma
WBCs: white blood cells
